# Health-related quality of life in children and adolescents undergoing intraoperative device closure of isolated perimembranous ventricular septal defects in southeastern China

**DOI:** 10.1186/s13019-019-1040-6

**Published:** 2019-12-16

**Authors:** Jiang-Shan Huang, Shu-Ting Huang, Kai-Peng Sun, Zhi-Nuan Hong, Liang-Wan Chen, Yur-Ren Kuo, Qiang Chen

**Affiliations:** 1 Department of Cardiovascular Surgery, Union Hospital, Fujian Medical University, Fuzhou, 350001 People’s Republic of China; 2 Department of Cardiac Surgery, Fujian Provincial Maternity and Children’s Hospital, affiliated hospital of Fujian Medical University, Fuzhou, 350001 People’s Republic of China; 3 Department of Surgery, Division of Plastic Surgery, Kaohsiung Medical University Hospital, 100 TzYou 1st Rd, Kaohsiung City, 80756 Taiwan

**Keywords:** Health-related quality of life, Children, Adolescents, Ventricular septal defect, Intraoperative device closure

## Abstract

**Objectives:**

To investigate the health-related quality of life (HRQOL) in children and adolescents who underwent intraoperative device closure of perimembranous ventricular septal defects (pmVSDs).

**Methods:**

From October 2017 to January 2018, a total of 126 children and adolescents with isolated pmVSDs who underwent intraoperative device closure were enrolled in this study. We used the Pediatric Quality of Life Inventory Measurement Models 4.0 generic core scales to measure HRQOL 24 h before the operation and three months and one year after surgery.

**Results:**

A total of 126 patients were successfully occluded. No severe complications occurred during the operative time, and 1 case of Mobitz type I atrioventricular block (AVB) and 1 case of complete cAVB occurred during the postoperative period. Compared with the data before the operation, the total score and five domain scores, including physical, psychosocial, emotional, social and psychological functioning, were significantly higher at three months after the operation. In addition, the total score, physical functioning score, and the psychosocial functioning score at the 1-year follow-up were even higher than those at 3 months after the operation.

**Conclusions:**

The present study suggests that intraoperative device closure of pmVSD could improve health-related quality of life in children/adolescents and that the improvement may progress as the time after the operation increases. Further studies should concentrate on comparisons with other medical methods, larger samples, and longer follow-up periods.

## Introduction

Ventricular septal defect (VSD) is one of the most common congenital cardiac defects in children and adolescents [1]. Surgical repair of VSD with cardiopulmonary bypass (CPB) has been a routine procedure for most patients with VSD, providing direct visual access to the defects. However, surgical repair is inevitably associated with many complications and surgical scars, which may impact neurodevelopmental, psychosocial, and physical functioning and diminish quality of life, especially for children and adolescents [2–7]. With the development of various occluders, transcatheter device closure of VSD has gradually become an alternative treatment and has been widely applied in the last decade, especially for perimembranous VSD. The transcatheter technique is acceptable for patients with an excellent rate of closure and only leaves a puncture on the surface, which offers an impressive cosmetic result [8, 9]. Recently, intraoperative device closure of VSD under the guidance of transesophageal echocardiography (TEE) has been widely and successfully used in China, and previous reports have already proven its feasibility and safety [10–12]. With regard to the incidence of a particular functional impairment in the cardiac population, health-related quality of life (HRQOL) has already been used as an essential assessment of cardiac operations [13–15]. A literature search did not reveal any report focusing on HRQOL after intraoperative device closure of pmVSD in children and adolescents. The present study aimed to explore HRQOL before and after intraoperative device closure of pmVSD in children and adolescents using the Pediatric Quality of Life Inventory (PedsQLTM) 4.0 generic core scale as an evaluation tool.

## Materials and methods

### Patients

The participants were enrolled in our cardiac department from October 2017 to January 2018. The inclusion criteria were as follows: restrictive perimembranous VSD, clinical and ECG evidence of significant left-to-right shunts and isolated VSD with the size of VSD ranging from 3 to 7 mm, and (or) a certain degree of heart chamber enlargement and (or) mild to moderate pulmonary hypertension. The exclusion criteria included the following: nonrestrictive VSD; size of VSD larger than 8 mm or smaller than 2 mm, especially those with large VSD; and high pulmonary hypertension coexisting other cardiac anomalies. Children or adolescents who had a developmental disability, intelligence defect or other condition that might affect HRQOL were also excluded from this study. The preoperative baseline characteristics of patients enrolled in this study are shown in Table 1.

**Table 1:** Preoperative baseline characteristics

Characteristic	Patients enrolled in this study
Number	126
Age(years)	5.2 ± 2.3
Sex(male/female)	76/50
Family income,n(%)	
Poor	20(15.8%)
Median level	72(57.4%)
Good	34(27.0%)
VSD size, mm	5.3 ± 1.6
Occluder size, mm	6.8 ± 1.5
Diameter of the sheath	5-8F

### Operative technique

TEE was used to assess the VSD position and the circumferential margins, especially their relationship with the aortic valve and tricuspid valve. A minimal incision (2–3 cm) was made through a lower partial median sternotomy. The pericardium was suspended to expose the right ventricle. The puncture site of the right ventricle was determined by indenting the free wall under TEE guidance to ensure that the puncture site was perpendicular to the defect and free of internal cardiac tissue. The guide wire was introduced into the right ventricle and then through the VSD defect to the left ventricle. A delivery sheath was introduced into the left ventricle through the guide wire to establish a delivery pathway. Then, the wire and the inner dilator were removed. The occluder was loaded into the delivery sheath with the help of a loading cable. Then, the occluder was advanced to the tip of the sheath, and the left disc was first deployed followed by the right disc. TEE assessment was performed to detect residual shunt or valve dysfunction [10–12].

### Instruments

We used the PedsQLTM 4.0 generic core scale to assess health-related QOL in this study. This instrument evaluates five aspects: physical functioning, psychological functioning, emotional functioning, social functioning, and school functioning. We rated the items on a five-point linear scale among participants aged 8–18 years (0 = never a problem; 1 = almost never an issue; 2 = sometimes a problem; 3 = often a problem; 4 = almost always a problem) and on a 3-point scale among participants aged 5–7 years (0 = not at all a problem; 2 = sometimes a problem; 3 = always a problem). Items of this instrument were reverse scored and linearly transformed into a 0–100 scale. A higher score suggests better HRQOL.

### Procedure

Two independent cardiac doctors were responsible for this investigation. We obtained the demographic and operation data of the participants through the electronic medical database. For the measurement of HRQOL changes before and after intraoperative device closure of pmVSD, the investigation was conducted at three time points: 24 h before the operation and 3 months and 1 year after the surgery. The first investigation was conducted in the meeting room, and the other two were performed at the hospital outpatient department when patients returned for physical examination. For participants aged 5–7 years old, the interviewer asked about the frequency of problems in the past month and filled in the form. For participants aged 8–14 years old, participants filled in the questionnaire alone.

### Statistical analysis

Continuous variables are expressed as x ± s; the t-test or analysis of variance was applied for continuous variables, and the χ^2^ or Fisher’s test was applied for categorical variables. A *p* value < 0.05 was defined as statistically significant. We used SPSS 17.0 software (SPSS Inc., Chicago, Illinois, United States of America) to analyze the data.

## Results

In total, 126 patients were successfully closed by undergoing intraoperative device closure of pmVSD. No patients were required to undergo surgical repair. Follow-up items included cardiac function, TTE, and electrocardiograph (ECG). Only 1 case of Mobitz type I atrioventricular block (AVB) and 1 case of late-onset complete AVB were recorded. A permanent pacemaker was installed in this patient with complete AVB. No other severe complications, including death, device disruption or failure, endocarditis, thrombosis, aortic valve distortion and permanent rhythm disturbances, occurred in the hospital stay or during 12-month follow-up period. The details of the follow-up results are shown in Table 2.

**Table 2:** Post-operative and follow-up complication results

Complication type	Time after intraoperative device closure		
	Hospitalization	3 months	1 year
Cerebrovascular accident	0	0	0
Small residual shunt	8	0	0
Large residual shunt	0	0	0
Transient Arrhythmia	5	1	1
Complete AVB	0	1	0
Mobitz type I AVB	1	0	0
Device embolization/failure	0	0	0
Low cardiac output syndrome	0	0	0
Pulmonary infection	2	0	0
Delayed healing of wound	1	0	0
Pericardial effusion	2	0	0
Pleural effusion	1	0	0

The health-related QOL change and comparison results are presented in Table 3. Compared with scores before surgery, the total score and the scores of the five domains, physical functioning, psychosocial functioning, emotional function, social functioning, and psychological functioning, were significantly higher at 3 months after the operation (*P* < 0.05). In addition, the total score, physical functioning score, and psychosocial functioning score were even higher 1 year after the operation (*P* < 0.05).

**Table 3:** Score change and comparison of health-related QOL

Domains	Before operation	3 months	1-year
Total score	66.1 ± 15.3	75.0 ± 11.4^#^	79.2 ± 9.7^*^
Physical functioning	68.4 ± 18.6	83.5 ± 11.6^#^	87.5 ± 8.8^*^
Psychosocial functioning	64.9 ± 16.1	70.4 ± 14.1^#^	74.8 ± 12.9^*^
Emotional functioning	56.1 ± 18.9	66.9 ± 17.7^#^	71.1 ± 16.4
Social functioning	74.5 ± 21.9	77.5 ± 19.7^#^	81.5 ± 16.7
School functioning	64.1 ± 19.1	66.9 ± 19.3^#^	71.8 ± 18.5

# means *P* < 0.05 compared with before operation, * means *P* < 0.05 compared with 3 months after operation

## Discussion

A ventricular septal defect is the most common congenital cardiac defect. Surgical repair under CPB was considered the gold-standard treatment for the closure of VSD, but this method remained limited by cosmetic problems and the potential risk of CPB [16, 17]. Transcatheter device closure of VSD showed a promising result and mid-term follow-up in recent reports [8, 9]. Yang and his colleagues reported a series of 848 patients with perimembranous VSD undergoing transcatheter device closure with a success rate of 98.1%, and there were only two cases of cAVB requiring pacemaker implantation during follow-up. The authors concluded that transcatheter pmVSD closure could be performed safely and successfully with low morbidity and mortality [15]. Wang L and coworkers reported a series of 525 children (aged between 2 and 12 years) with pmVSD who underwent transcatheter device closure. The successful device closure rate was 95.6%, and a total of three major adverse events (0.6%) were reported [18]. Compared with a conventional surgical procedure, transcatheter device closure only leaves a puncture and does not require CPB, preventing patients from myocardial reperfusion injury and resulting in a rapid recovery. However, transcatheter device closure was limited by potential vascular injury and exposure to X-rays. There were also technical challenges when applied to infants or patients with a weight below 10 kg [19]. Under these conditions, intraoperative device closure of VSD developed from the above two treatments. Xing and his colleagues reported a series of 458 patients undergoing minimally invasive transthoracic device closure of VSD with a success rate of 96.29% and no severe complications or death during follow-up in 2015 [10]. Our report also showed that a series of 1033 patients were successfully occluded with this procedure with little difficulty. We concluded that the intraoperative device closure of VSD on a beating heart was a safe and feasible alternative to conventional surgical repair and transcatheter device closure [20]. The comparisons among surgical repair with CPB, intraoperative device closure and transcatheter device closure of VSD regarding risks and benefits were summarized in table 4.^12^(the data derived from our team’s previous report).

**Table 4:** The comparisons among surgical repair, intraoperative device and transcatheter device closure of VSD regarding risks and benefits based on the previous report

	Surgical repair	Intraoperative device closure	Transcather device closure
CPB(min)	56.6 ± 13.5	/	/
The incision length(cm)	11.8 ± 2.1*	3.1 ± 1.2	/
Hospital stay(d)	8.5 ± 3.4*	4.2 ± 1.6	3.9 ± 2.2
Hospital costs (10,000 RMB)	5.53 ± 0.82*	3.12 ± 0.25	3.22 ± 0.43
Hospital death	0/86	0/90	0/71
cAVB	0/86	1/90	0/71
PPM	0//86	0/90	0/71
Brain damage	0/86	0/90	0/71

*CPB* Cardiopulmonary bypass, *cAVB* Complete atrioventricular block, *PPM* Permanent pacemaker, *RMB* Renminbi. *:*p* < 0.05

In this study, our follow-up results were consistent with previous reports. A total of 126 patients were all successfully occluded, and no patient was required to undergo surgical repair. Complete AVB is a significant complication for device closure of VSD and may significantly affect the quality of life of patients, but the exact mechanism remains unclear. This may result from the different understanding of cardiac anatomy among operators and the distance of the delivery pathway. The incidence of transient cAVB ranged from 1 to 5% in patients undergoing transcatheter device closure of VSD [21–23]. Only 1 case of late-onset complete AVB occurred during the follow up, and a permanent pacemaker was inserted into this patient. We attribute this impressively low incidence of cAVB to the following two reasons: First, this procedure provided a shorter and direct distance of delivery pathway causing less mechanical trauma/compression and second, we chose a suitable size of the device for each patient, thus minimizing pressure on the tricuspid annulus where the AV node lies. This study also demonstrated that intraoperative device closure was a safe and feasible alternative for select patients with pmVSD.

A literature search was unable to identify any reports that focused on the HRQOL of patients who underwent intraoperative device closure for pmVSD. The investigation of HRQOL in children and adolescents with congenital VSD would provide complementary information for the decisions of health professionals. In this study, we used the Pediatric Quality of Life Inventory Measurement Models 4.0 generic core scales to measure the health-related QOL. Since Varni JW and his coworkers first proposed the Pediatric Quality of Life Inventory Measurement Models 4.0 generic core scale in 2001, this instrument has been widely used and proven to be reliable and valid [24]. Matza and coworkers emphasized when choosing a questionnaire that the minimum age that children can answer questions about quality of life and the format of the instrument suitable for the level of understanding of each age group should be taken into consideration [25]. Varni et al. reported in 2007 that children as young as 5 years old can reliably and validly self-report their HRQOL [26]. In addition, the PedsQL™ 4.0 Generic Core Scales have already been widely applied in evaluating the quality of life of patients with congenital heart disease [27, 28]. Hao Y and his colleagues proposed a Chinese version of the Pediatric Quality of Life Inventory 4.0 generic core scales and concluded that this instrument had acceptable psychometric properties with the exception of the construct validity tested by confirmatory factor analysis and the internal reliability for self-report [29].

Thus, we chose this instrument in our study and assumed that intraoperative device closure could improve HRQOL in children/adolescents with isolated pmVSD. We found that the total score and the five domain scores, physical, psychosocial, emotional, social and emotional functioning, were significantly higher at the three-month follow-up compared with the scores before the operation (*P* < 0.05). Such results indicated that the patient’s quality of life was significantly improved in the short term after surgery. However, the scores for emotional functioning, social functioning, and school functioning were not significantly higher than the scores at 12 months after the operation. However, the total score, physical functioning score and psychosocial functioning score at 12 months were all even higher than the scores at 3 months after the operation. This encouraging trend suggested that intraoperative device closure of pmVSD on a beating heart can improve health-related QOL in children/adolescents, and this improvement seems to progress as time goes on. We contributed this improvement to the lasting improvement in cardiac function and the low complication and high closure rate of intraoperative device closure of pmVSD in our institution. Satisfactory surgical results also significantly improved the patient’s quality of life. However, it is not enough to conclude whether this improvement would continue and how long it could last. We could not analyze the subpopulation to identify high-risk factors that could affect postoperative HRQOL due to the limited sample size in this study. Patients undergoing intraoperative device closure of pmVSD deserve more attention for their prognosis and quality of life.

The following factors limited this study. First, this study had a small sample size and was only conducted in one institution. Second, the follow-up period lasted only 12 months. A larger sample and longer follow-up are required for a future study. Third, there was no control group involving children/adolescents with isolated VSD undergoing other treatments, including transcatheter device closure or surgical repair, to which to compare the results. Thus, in a future study, to elucidate the impact of intraoperative device closure of VSD on HRQOL, a control group would be needed.

## Conclusion

In conclusion, intraoperative device closure of pmVSD on a beating heart can improve health-related QOL in children/adolescents. The improvement of QOL increased as time passed during the follow-up period. Further studies should focus on the comparison between patients undergoing intraoperative device closure of pmVSD with patients with VSD undergoing other treatments or patients with VSD who did not undergo therapy.

## Data Availability

Data sharing not applicable to this article as no data sets were generated or analyzed during the current study.

## Abbreviations

AVB: Atrioventricular block
CPB: Cardiopulmonary bypass
HRQOL: Health-related quality of life
TEE: Transesophageal echocardiography
VSD: Ventricular septal defects
